# Epigenetic modulation of the tumor immune microenvironment by nanoinducers to potentiate cancer immunotherapy

**DOI:** 10.20892/j.issn.2095-3941.2021.0514

**Published:** 2021-09-27

**Authors:** Ning Zhang, Xishan Hao

**Affiliations:** 1 Translational Cancer Research Center, Peking University, Peking University First Hospital, Beijing 100034, China; 2 Tianjin Medical University Cancer Institute and Hospital, National Clinical Research Center for Cancer, Key Laboratory of Cancer Prevention and Therapy, Tianjin, Tianjin’s Clinical Research Center for Cancer, Tianjin 300060, China

Epigenetic dysregulation is a key factor leading to oncogenesis and tumor progression^1^. To date, seven agents in three epigenetic target classes have been approved by the U.S. Food and Drug Administration for the treatment of diverse malignancies. Despite this approval for clinical application, the mechanisms of many epigenetic modulators are not completely understood, because many genes are epigenetically regulated. Recent studies have revealed that transcriptional activation of retroelements in the genome is a mechanism associated with the anti-tumor activity of epigenetic modulators. Retroelement transcription leads to intracellular accumulation of dsRNA, which binds and stimulates pattern recognition receptors, which in turn trigger the production of type I interferons (IFNs)^2^. Autocrine and paracrine IFN-α/β signaling in the tumor microenvironment promotes the secretion of other pro-inflammatory cytokines and chemokines, which enhance anti-tumor immunity by increasing antigen presentation and immune cell infiltration^3^. However, pro-inflammatory cytokines such as IFNs also upregulate multiple immune checkpoints, thereby leading to immune evasion in many cancers such as melanoma and breast cancer. These contradictory effects limit the anti-tumor activity of epigenetic modulators. Another obstacle in epigenetic therapy is the poor tumor selectivity of the agents. Because the targets of epigenetic agents are prevalently expressed throughout the body, off-tumor drug deposition inevitably leads to systemic toxicity, such as bone marrow suppression, central nervous toxicity, or systemic inflammation.

Given the limited efficacy of epigenetic therapy as a monotherapy, its combination with other treatments is being extensively explored in clinical settings^4^. Accumulating evidence supports the use of a combination of epigenetic therapy with chemotherapy, radiation therapy, molecularly targeted therapy, and immunotherapy. For instance, combination use of the histone methyltransferase G9a inhibitor CM-272 with cisplatin or an immune checkpoint inhibitor significantly suppresses tumor metastasis in a mouse model. However, combination therapy with two drugs in their free forms usually causes more severe adverse effects, owing to off-tumor exposure. More rational combination strategies and target delivery formulations would be beneficial^5^.

**Figure 1:**
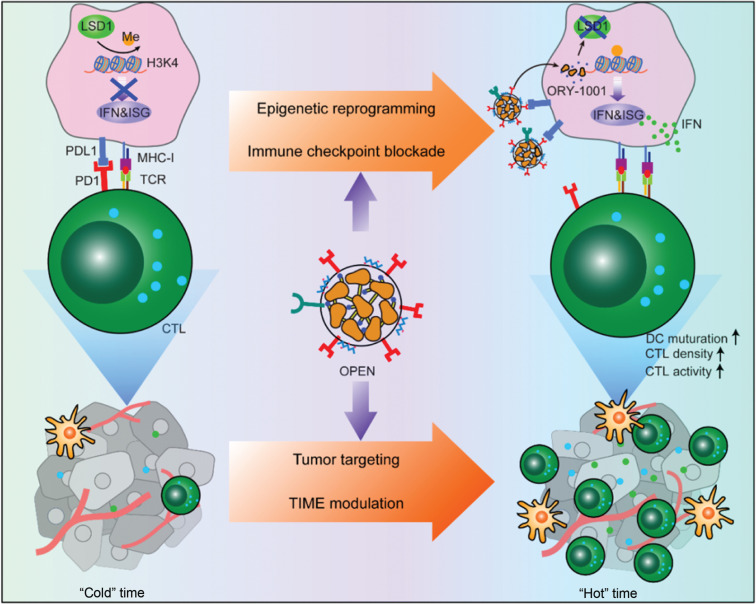
Schematic illustration of OPEN for epigenetic modulation and immune checkpoint blockade.

Recently, Prof. Yaping Li and colleagues^6^ (Shanghai Institute of Materia Medica, CAS, China), in *Nature Nanotechnology*, have reported a new strategy to improve the specificity of epigenetic drugs by using an engineered T lymphocyte membrane camouflage strategy (**Figure 1**). In their work, ORY-1001, an inhibitor of lysine-specific histone demethylase 1 (a nuclear enzyme that demethylates mono- and dimethylated lysine 4 of histone H3), was encapsulated in programmed death receptor 1-displaying nanovesicles called OPEN. The OPEN bind programmed death receptor ligand 1 (PDL1) on cancer cells and enable cell specific delivery of ORY-1001, thereby inducing the accumulation of mono- and dimethylated H3K4. Epigenetic agents upregulate the expression of IFNs and downstream IFN-stimulated genes such as major histocompatibility complex I (MHC-I) and PDL1. Upregulated MHC-I improves antigen display, thus facilitating cancer cell recognition by T lymphocytes. The increased PDL1 is blocked by subsequent OPEN treatment. Compared with free ORY-1001, OPEN increase intra-tumor drug accumulation and decreases liver drug exposure after intravenous injection, thereby alleviating the side effects of ORY-1001. Intra-tumoral ORY-1001 reshapes the tumor immune microenvironment (from “cold” to “hot”) by increasing the density of mature dendritic cells, proliferation of cytotoxic T lymphocytes, and antigen display of cancer cells. Consequently, OPEN significantly inhibit the growth of primary tumors and prolongs survival in several tumor animal models.

This work, the first example of an epigenetic nanomodulator, successfully addresses two major hurdles of epigenetic therapy: non-specific accumulation and the stimulation of both anti-tumoral and pro-tumoral activities. The unique design of the epigenetic nanomodulator utilizes an engineered T lymphocyte membrane to achieve tumor-targeted delivery of epigenetic drugs and immune checkpoint blockade. An intriguing property of OPEN is that they can replenish their own binding receptors, thus avoiding receptor depletion-induced desensitization. Given that all materials used for the construction of OPEN are biocompatible, and the preparation techniques are achievable in industrial settings, this platform has great potential for clinical translation. This work represents substantial progress in the effective combination of epigenetic therapy and immunotherapy for the treatment of cancer.
